# The reactome pathway knowledgebase

**DOI:** 10.1093/nar/gkz1031

**Published:** 2019-11-06

**Authors:** Bijay Jassal, Lisa Matthews, Guilherme Viteri, Chuqiao Gong, Pascual Lorente, Antonio Fabregat, Konstantinos Sidiropoulos, Justin Cook, Marc Gillespie, Robin Haw, Fred Loney, Bruce May, Marija Milacic, Karen Rothfels, Cristoffer Sevilla, Veronica Shamovsky, Solomon Shorser, Thawfeek Varusai, Joel Weiser, Guanming Wu, Lincoln Stein, Henning Hermjakob, Peter D’Eustachio

**Affiliations:** 1 Ontario Institute for Cancer Research, Toronto, ON M5G0A3, Canada; 2 NYU School of Medicine, New York, NY 10016, USA; 3 European Molecular Biology Laboratory, European Bioinformatics Institute (EMBL-EBI), Wellcome Genome Campus, Hinxton, Cambridgeshire, CB10 1SD, UK; 4 Open Targets, Wellcome Genome Campus, Hinxton, Cambridgeshire, CB10 1SD, UK; 5 College of Pharmacy and Health Sciences, St. John's University, Queens, NY 11439, USA; 6 Oregon Health and Science University, Portland, OR 97239, USA; 7 Department of Molecular Genetics, University of Toronto, Toronto, ON, M5S 1A1, Canada; 8 National Center for Protein Sciences, Beijing, China

## Abstract

The Reactome Knowledgebase (https://reactome.org) provides molecular details of signal transduction, transport, DNA replication, metabolism and other cellular processes as an ordered network of molecular transformations in a single consistent data model, an extended version of a classic metabolic map. Reactome functions both as an archive of biological processes and as a tool for discovering functional relationships in data such as gene expression profiles or somatic mutation catalogs from tumor cells. To extend our ability to annotate human disease processes, we have implemented a new drug class and have used it initially to annotate drugs relevant to cardiovascular disease. Our annotation model depends on external domain experts to identify new areas for annotation and to review new content. New web pages facilitate recruitment of community experts and allow those who have contributed to Reactome to identify their contributions and link them to their ORCID records. To improve visualization of our content, we have implemented a new tool to automatically lay out the components of individual reactions with multiple options for downloading the reaction diagrams and associated data, and a new display of our event hierarchy that will facilitate visual interpretation of pathway analysis results.

## INTRODUCTION

At the cellular level, life is a network of molecular reactions that enable signal transduction, transport, DNA replication, protein synthesis and intermediary metabolism. A variety of online resources capture aspects of this information at the level of individual reactions such as Rhea (1) or at the level of reaction sequences spanning various domains of biology such as KEGG (2), MetaCyc (3) or PANTHER (4). The Reactome Knowledgebase is distinctive in focusing its manual annotation effort on a single species, *Homo sapiens*, and applying a single consistent data model across all domains of biology. Processes are systematically described in molecular detail to generate an ordered network of molecular transformations, resulting in an extended version of a classic metabolic map (5,6). The Reactome Knowledgebase systematically links human proteins to their molecular functions, providing a resource that functions both as an archive of biological processes and as a tool for discovering novel functional relationships in data such as gene expression studies or catalogs of somatic mutations in tumor cells.

Reactome (version 70—September 2019) has entries for 10 867 human protein-coding genes, 53% of the 20 454 predicted human protein-coding genes (Ensembl release 97—July 2019—http://www.ensembl.org/Homo_sapiens/Info/Annotation), supporting the annotation of 25 849 specific forms of proteins distinguished by co- and post-translational modifications and subcellular localizations. These function with 1856 naturally occurring small molecules as substrates, catalysts and regulators in 11 638 reactions annotated on the basis of data from 30 398 literature references. These reactions are grouped into 1803 pathways (e.g. interleukin-15 signaling, phosphatidylinositol phosphate metabolism and receptor-mediated mitophagy) grouped into 26 superpathways (e.g. immune system, metabolism and autophagy) that describe normal cellular functions. Notable recent additions include extended annotations of SUMOylation and NEDDylation reactions and their regulatory consequences, annotations of NOTCH and RUNX signaling processes, systematic annotation of the processes of autophagy, and annotation of the metabolism of arachidonate-derived proresolvin mediators.

An additional ‘disease’ superpathway groups 484 annotations of disease counterparts of these normal cellular processes. These disease annotations include 1599 variant proteins and their post-translationally modified forms derived from 308 gene products, used to annotate 970 disease-specific reactions, tagged with 387 Disease Ontology terms (7).

Notable recent changes in Reactome include expanding the scope of the project to support annotation of the molecular function of drugs, developing new tools to facilitate community participation in annotation and to explicitly acknowledge it, and developing new web features to improve the layout of individual reactions and the visualization of our event hierarchy.

## ANNOTATING MOLECULAR MECHANISMS OF DRUG ACTION

A ‘drug’ is not a molecularly distinct kind of physical entity but rather a role that the entity can assume under specific circumstances. For Reactome, a drug is a physical entity not normally present in a human system and not a normal dietary constituent that when introduced into the system interacts with the naturally occurring components of the system to modulate their molecular functions. A new ‘drug’ class of physical entities in our data model distinguishes chemical drugs (e.g. β-blockers) from protein drugs (e.g. therapeutic antibodies) and RNA drugs (e.g. synthetic small RNAs).

**Figure 1.**
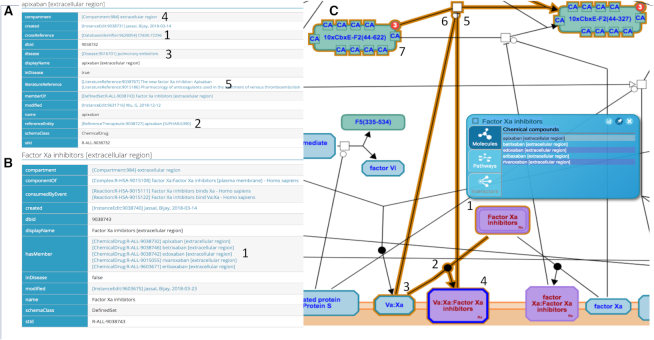
Annotating drugs: antithrombotic activity of Factor Xa inhibitors. (**A**) The Reactome annotation of apixaban associates it with reference information in the ChEBI (1) and IUPHAR (2) databases, with a disease process, pulmonary embolism, from the Disease Ontology (3), with a cellular component, ‘extracellular region’ from GO (4), and with literature references from PubMed (5). Each of these entries is associated with a Reactome identifier; following the link on the Reactome page takes the user to the web page for that entry in the reference database. (**B**) Apixiban is grouped with four other similarly annotated small molecules (1), betrixaban, endoxaban, reviroxaban and eribaxan, to form the set of Factor Xa inhibitors. (**C**) The shared molecular function of this set of molecules (1) is represented by annotating their binding (2) to the complex of Factors Va and Xa (3) to form an inactive complex (4), thereby negatively regulating (5) the cleavage (6) of Factor 2 (7). In our iconography, entities with a drug role are shown with their usual icon shapes but shaded purple and with an ‘Rx’ tag in the lower right corner of the icon.

As shown for the antithrombotic chemical apixaban in Figure 1A, each chemical drug instance is mapped to its counterpart in IUPHAR (8) and if one is available in ChEBI (9) and for additional pharmacological data. The drug instance is also associated with a disease target using terms from the Disease Ontology (7) and a subcellular location using terms from the GO cellular component ontology (10). When several such drugs form a chemically related family with a single target and mechanism of action, we group them into a set (Figure 1B); that set is then used to create reactions to annotate the shared action (either negative or positive) of the set members on the target. In the case of apixaban and closely related chemical drugs that bind and inhibit Factor Xa both alone and as a complex with Factor Va, a reaction shows drug binding to the complex to form a drug:protein complex that negatively regulates cleavage of Factor II (Figure 1C).

Our September 2019 release includes annotations of effects of 222 drugs, mostly chemical drugs in widespread use to treat thrombosis and other cardiovascular diseases. Work is underway to extend annotations to drugs involved in other disease processes, and to other stages of the complete drug life cycle, linking the molecular function of its active form to reactions for its uptake, its activation if it enters the body in prodrug from, and its inactivation and elimination.

Of all the exogenous molecules that can perturb human biological processes, which ones qualify as drugs? A molecule in widespread clinical use as indicated by its IUPHAR annotations is clearly qualified. In addition, we include molecules whose potential therapeutic mechanisms of action have been defined at a molecular level even if those molecules are not yet approved for clinical use or are restricted to a pre-clinical setting because of toxic side effects, if this annotation is useful to illustrate the mechanism of action and possible limitations in the application of a drug class in human systems. These broad boundaries are compatible with Reactome’s role as a biomedical research tool, rather than as a resource to support clinical decision making.

Drug annotation to date centers on cases in which a drug binds a protein (normal or genetic variant) and alters the protein’s default function, thereby regulating it. The Reactome data model can accommodate more diverse and complicated drug–target interactions, and can likewise accommodate the other stages of a drug’s life cycle in the body in which it is taken up, transported to a target site, activated, then inactivated and excreted. These extensions are the focus of work now getting underway.

## FACILITATING COMMUNITY INVOLVEMENT IN ANNOTATION

Domain experts participate in creating, validating and updating Reactome annotations at all levels of granularity from the details of an individual reaction to the organization of a superpathway. We have always solicited such participation, and in several cases have organized formal collaborations to annotate a subject area. To broaden the process to ensure that it is open to all interested biologists, we have implemented two new features on our web site, one to enable new users to participate in the review process, and one to enable individuals who have already contributed to Reactome to associate the Reactome events on which they have worked with their ORCID records (https://orcid.org).

**Figure 2.**
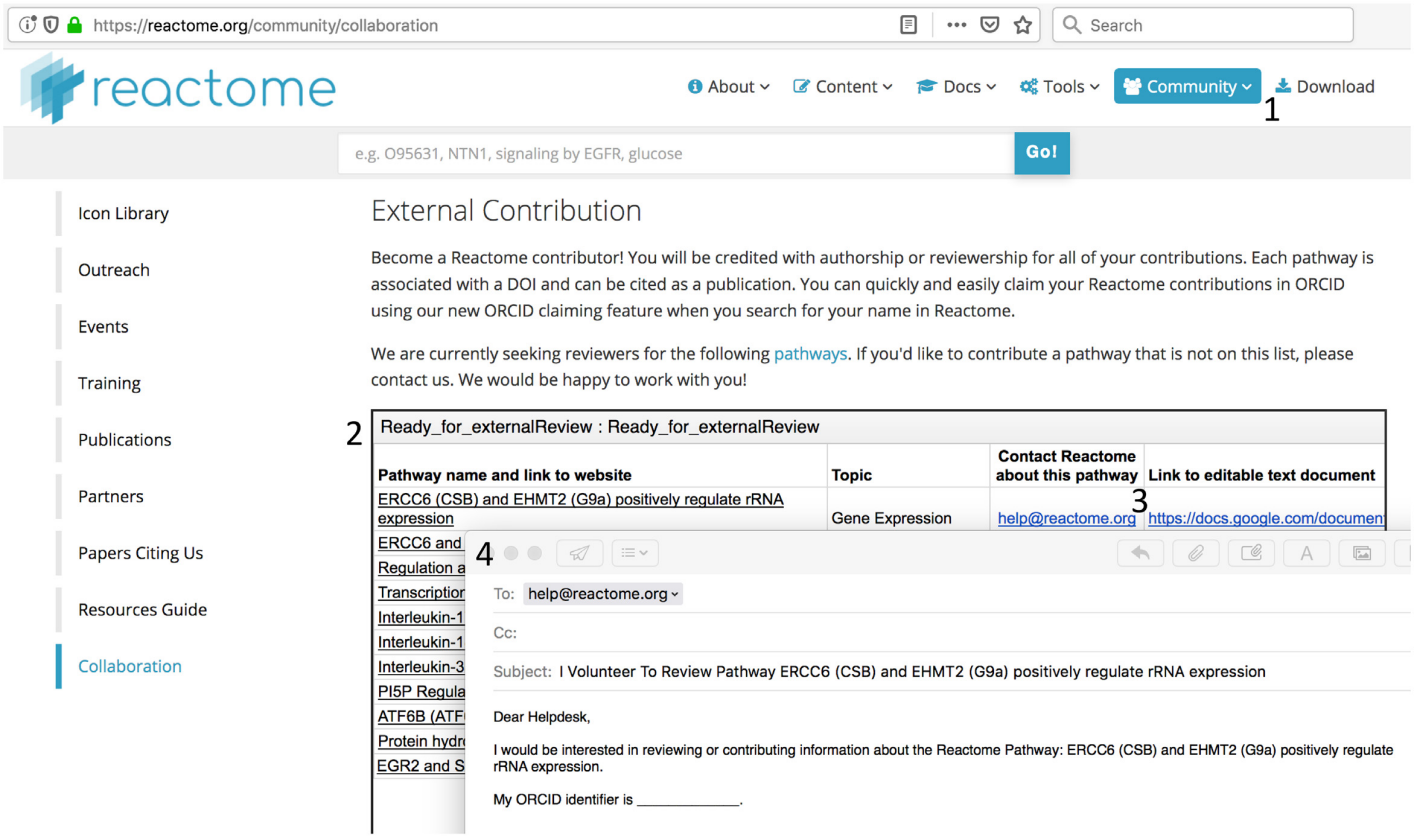
Soliciting community input for new annotations. The new ‘external contribution’ web page is accessible via the community tab (1) at the top of the Reactome homepage. It displays a list of newly annotated, internally reviewed events for which we are seeking external reviews (2). Clicking on the help@reactome.org link next to an event of interest (3) opens an e-mail message (4) pre-populated with the event’s details with slots to accommodate personal details. Clicking on the event itself in the table opens a Google Document that contains a text description of the event.

Community participation is enabled by a web page (Figure 2) that lists all newly created events that are ready for final review, with an on-line form that allows a person to identify an event of interest and volunteer to work with us on it. The page also can be used to propose new topics for annotation.

**Figure 3.**
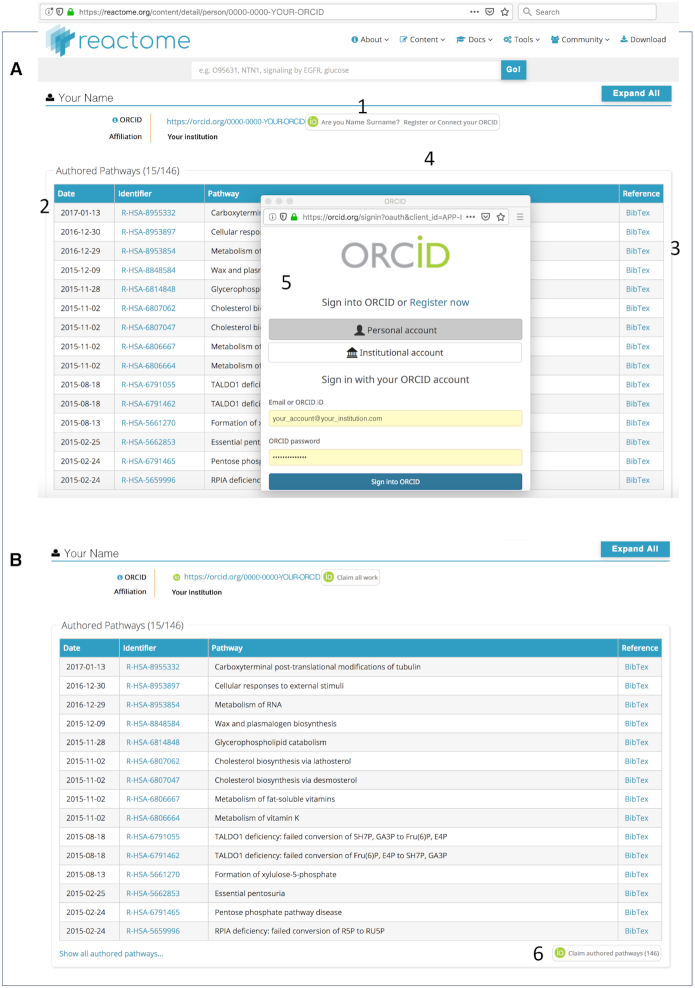
Associating expert authors’ and reviewers’ ORCID IDs with Reactome events. (**A**). A search (1) on a person’s name returns a list (2) of all events (pathways and reactions) with which that person is associated as author or reviewer. Details of an individual event can be exported in BibTex (www.bibtex.org) format (3). Clicking on the ‘Are you [person]?’ text at the top of the event list opens a window in which the person can enter his or her ORCID ID and password (4). These credentials are verified and the web page that lists the events is displayed (**B**) with buttons (5) that enable the person to claim authored and reviewed pathways and reactions.

To associate a person’s ORCID identifier with events on which the person has worked, the Reactome search engine has been modified so that a search on the person’s name now returns a web page that lists all events (reactions and pathways) to which the named person has contributed as an author or reviewer (Figure 3). Features on that page enable the person to examine individual events, to download citations to them in BibTex format, and to validate his or her ORCID identifier and then to claim those events to make them part of his or her ORCID record. When a person makes contributions over multiple Reactome release cycles, repetition of this claiming process will identify the new work and add it to the ORCID record.

## IMPROVED REACTION AND PATHWAY VISUALIZATION

### Automatic layout of individual reactions

**Figure 4.**
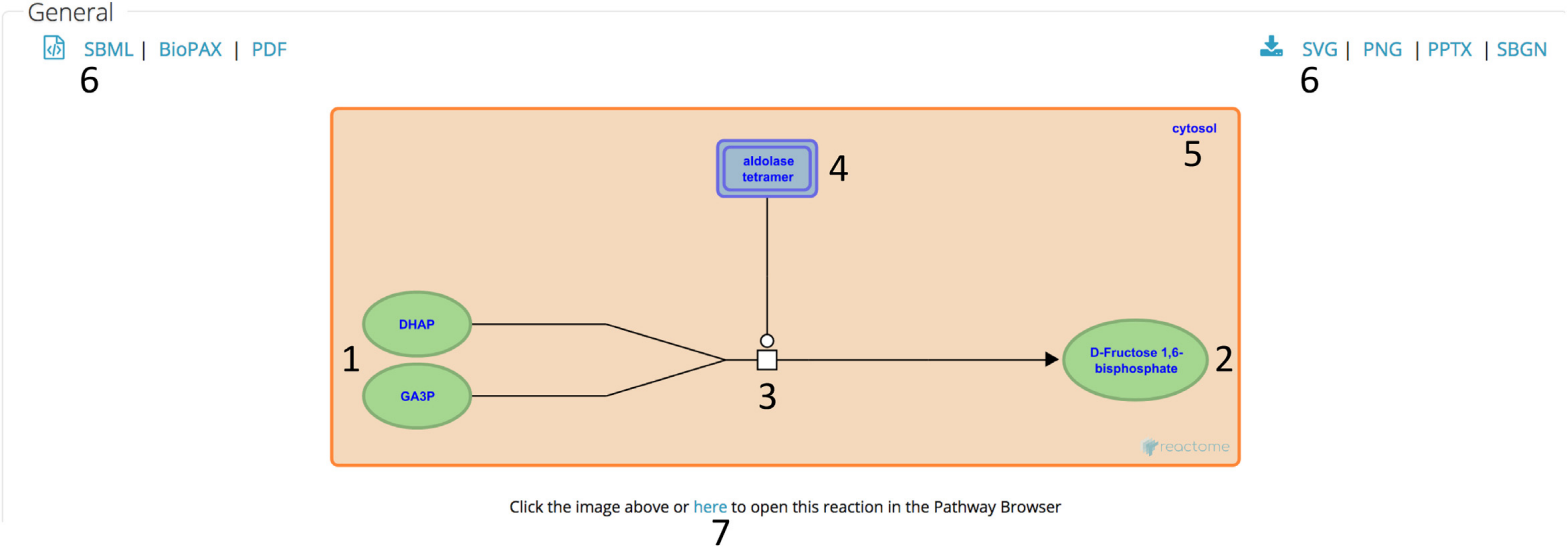
Automated reaction layout. When the details of a reaction are displayed, e.g., as the result of a search, a graphical representation of the individual reaction is generated that identifies its inputs (1), outputs (2), type (here, chemical transformation) (3), catalyst (4) and subcellular location or locations (5). The reaction image and its associated data can be exported in a variety of formats (6). Clicking on the image or the text below it takes the user to a view of the reaction in its pathway context (7).

Human usability of web searches that return one or more individual reactions, and of downloadable PDF files with text descriptions of all reactions and subpathways in a pathway is improved by providing a diagram of each reaction that shows its participants (inputs, outputs, regulators and catalyst) laid out in a conventional left-to-right mode, localized within the appropriate cellular compartments. When a reaction involves more than one compartment, e.g., signal transduction or transport, the compartments are correctly located with respect to one another and the participating entities. A pilot version of a script to generate such diagrams has been deployed on our web site (Figure 4). The resulting images and their associated data can be exported in a variety of formats.

### Alternative pathway visualization scheme

**Figure 5.**
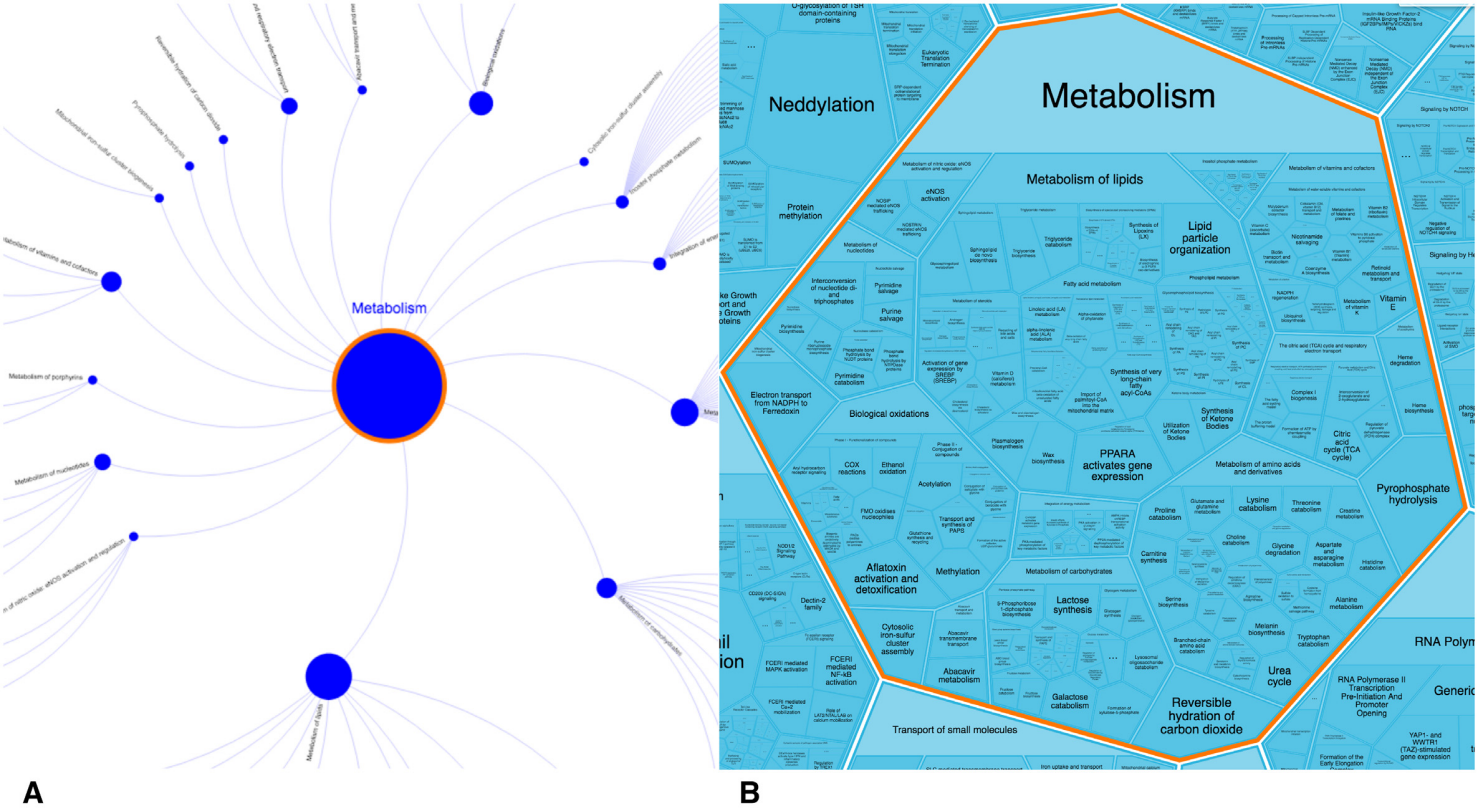
Pathway browser views of the ‘Metabolism’ superpathway and its children. (**A**). Standard ‘fireworks’ format. (**B**). Voronoi diagram (‘ReacFoam’) format.

The ‘fireworks’ view of our event hierarchy represents each superpathway as a node surrounded by concentric rings of nodes representing the superpathway’s child pathways, their child pathways and so on. Nodes are scaled in proportion to the numbers of events they contain. Arc edges represent the part_of relationships between pathways and subpathways; multiple part_of relationships for a single pathway are readily represented as multiple arcs (Figure 5A). This layout provides a legible view of a large event hierarchy with complex parent-child relationships and is easily edited to accommodate both new domains of knowledge (superpathways) and new material added to existing pathways. To maintain this legibility, however, nodes must be small and most of the space in the diagram must be left empty. As a result, colorizing the ‘fireworks’ view to display an overview of the results, e.g., of overlaying a gene expression dataset on the Reactome event hierarchy often yields results that are not readily viewed by a human user. To solve this display problem, we have developed an alternative display option based on Voronoi diagrams (Wikipedia. Voronoi diagram https://en.wikipedia.org/w/index.php?title=Voronoi_diagram&oldid=908392936 (accessed 26 August 2019)). The resulting pathway display (Figure 5B) is partitioned into contiguous regions, each corresponding to a pathway and grouped according to the relationships among pathways specified in the event hierarchy. This arrangement devotes maximum possible space in the diagram to the pathway nodes. Figure 5 shows our metabolism node, comparing its fireworks and Voronoi diagram layouts.

## ACCESS TO DATA AND SOFTWARE

Reactome is open-source. All original Reactome data are available in various formats from the ‘downloads’ page of our web site (https://reactome.org/download-data) and all software is available from our GitHub repository (https://github.com/reactome).

## CONCLUSIONS

The Reactome Knowledgebase of the molecular details of human biological processes continues to grow in size and scope, notably with the development of tools to annotate the roles of drugs in these processes to yield an integrated description of default human processes and their modulation by drugs. We have implemented a new web feature to recruit experts to participate in our review process and other aspects of curation. New visualization features provide high-quality, downloadable diagrams of individual reactions and an overview of all of our content in a format that should facilitate exploration of gene expression and similar data sets.
